# Few-photon color imaging using energy-dispersive superconducting transition-edge sensor spectrometry

**DOI:** 10.1038/srep45660

**Published:** 2017-04-04

**Authors:** Kazuki Niwa, Takayuki Numata, Kaori Hattori, Daiji Fukuda

**Affiliations:** 1 Quantum Optical Measurement Group, Research Institute for Physical Measurement, National Metrology Institute of Japan, National Institute of Advanced Industrial Science and Technology (AIST), Japan

## Abstract

Highly sensitive spectral imaging is increasingly being demanded in bioanalysis research and industry to obtain the maximum information possible from molecules of different colors. We introduce an application of the superconducting transition-edge sensor (TES) technique to highly sensitive spectral imaging. A TES is an energy-dispersive photodetector that can distinguish the wavelength of each incident photon. Its effective spectral range is from the visible to the infrared (IR), up to 2800 nm, which is beyond the capabilities of other photodetectors. TES was employed in this study in a fiber-coupled optical scanning microscopy system, and a test sample of a three-color ink pattern was observed. A red–green–blue (RGB) image and a near-IR image were successfully obtained in the few-incident-photon regime, whereas only a black and white image could be obtained using a photomultiplier tube. Spectral data were also obtained from a selected focal area out of the entire image. The results of this study show that TES is feasible for use as an energy-dispersive photon-counting detector in spectral imaging applications.

Spectral imaging allows us to obtain multiple types of information in accordance with differences in color signals^1^. In life science research and industry, multiple biomolecules are observed in spectral imaging and other applications to determine the dynamic molecular mechanisms behind the biological phenomena. In many cases, biomolecules are labeled with color, fluorescence, and/or chromic dye probes to enable the identification of different features. In other cases, natural color phenomena are analyzed. For example, spectral changes in plant chlorophyll are indicators of stress. To assess the ratios of materials of different colors, spectral elements such as gratings or color filters are employed in conventional optical systems to discriminate light signals based on their wavelengths. The light intensity through spectral elements is, however, decreased before arriving at each photodetector because the light is separated, absorbed, and/or reflected. In imaging optics, the focal area of observation is quite small, so the light intensity from the area is weak and is often not detectable. Even using sensitive photon-counting detectors widely applied in spectral imaging equipment, such as photomultipliers (PMTs), single-photon avalanche diodes (SAPDs), and electron-multiplying charge-coupled devices (EM-CCDs), light signals can easily decrease to less than the background level. Furthermore, the sensitive bands (wavelength ranges) of such detectors are not sufficiently wide. For example, the sensitive bands of PMTs are primarily limited to the visible light range; therefore, light signals with longer wavelengths are difficult to detect. Wider-band-range detectors are able to detect a wider variety of color probes. Therefore, highly sensitive and broad-band photodetectors can contribute to spectral imaging applications by allowing darker samples and a wider variety of probes to be detected and analyzed simultaneously.

One candidate is a highly sensitive superconducting detector, such as a transition-edge sensor (TES) or a superconducting nanowire single-photon detector (SNSPD). An SNSPD exhibits excellent performance in quantum photonics and biotechnological applications, such as singlet oxygen luminescence dosimetry^2^ and fluorescence correlation spectroscopy^3^. TES is increasingly being employed as a detector to obtain quantum information, which requires high detection efficiency and photon number-resolving capability at telecommunication wavelengths^4^. In addition, TES has the potential to be applied in biosciences because of its unique energy-dispersive spectral single-photon-detecting ability.

A TES is made of a superconducting film that functions as a thermometer within its resistive transition between the normal and superconducting states. The heat capacity of a TES is tuned to be sensitive so that the detector exhibits a significant temperature rise because of the absorbance of the small power deposition of a single photon, even in the near-IR region (800–1500 nm). Therefore, a single photon incident on a TES can be detected by monitoring the electrical resistance of the TES (Fig. 1). The transition temperature of a TES is in the sub-Kelvin region, which allows nearly zero dark-count (background) stimulation by thermal energy.

A TES is basically an energy-dispersive photon detector; therefore, an increase in the electrical resistance of the TES reflects both the energy and the wavelength of the absorbed photon, which can be determined simultaneously. Therefore, a TES determines the wavelength of each incident photon directly, without using any wavelength-dispersive spectral elements, such as grating, prisms, or color filters. Consequently, a TES can drastically improve the sensitivity of spectral measurements, which contributes to the performance of spectral imaging techniques.

Previously, we constructed TES detectors optimized for the near-IR and telecommunication wavelength (1550 nm) regions. We achieved 98% detection efficiency at a wavelength of 850 nm, which is, to the best of our knowledge, the highest detection efficiency ever achieved^4^. The rise and decay times of the response signals, which are the times required for the amplitude of the pulse to become 1/e of the peak value, are 30 ns and 200 ns, respectively. These response times extend a counting rate of up to approximately 10^6^ photons/s. Above this maximum counting rate, signals from two photons are likely to overlap. If two photons arrive at a TES within the rise time, they cannot be distinguished from a single photon signal. Two photons arriving outside the rise time but within the decay time can be distinguished, but it is difficult to resolve the energy of these photons precisely. At a few-photon counting rate, the probability of signals from two photons overlapping is sufficiently low.

Here, we report the first demonstration of energy-dispersive TES spectral photon measurement for few-photon color imaging. We employed a fiber-coupled TES sensitive to a wide-wavelength region from the visible to the IR range as a spectral photon detector for a scanning two-dimensional (2-D) microscope. An RGB-color image was successfully observed in the few-incident-photon regime.

## Results and Discussion

### TES spectral photon detection

**Figure 1:**
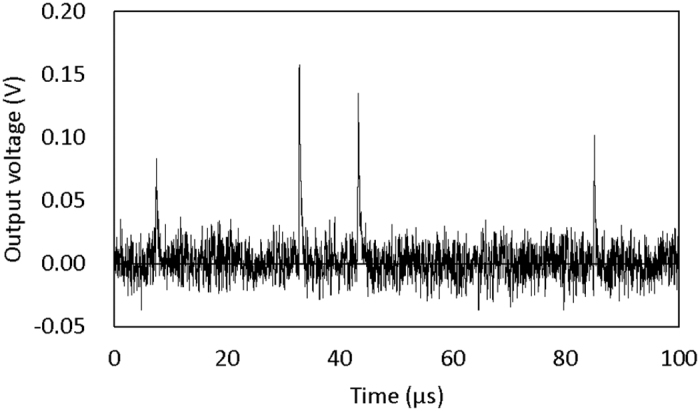
TES single-photon measurement. The incident photon is detected as a spike signal of output voltage that is proportional to electrical resistance of the TES. The height of the spike signal indicates the energy of incident photon, which gives the wavelength via the formula *E = hc/λ*. White noise, which is the background baseline noise of the output voltage reading, limits the spike signals of photons with too little energy to be detected and corresponds to the longest wavelength of the detectable photons.

By observing the electrical resistance of the TES, the incident energy of each photon can be detected as spike signals (Fig. 1). The height of each signal reflects the energy or wavelength via the formula *E = hc/λ*. The measured spectrum is constructed by plotting the number of photons against their wavelength.

**Figure 2:**
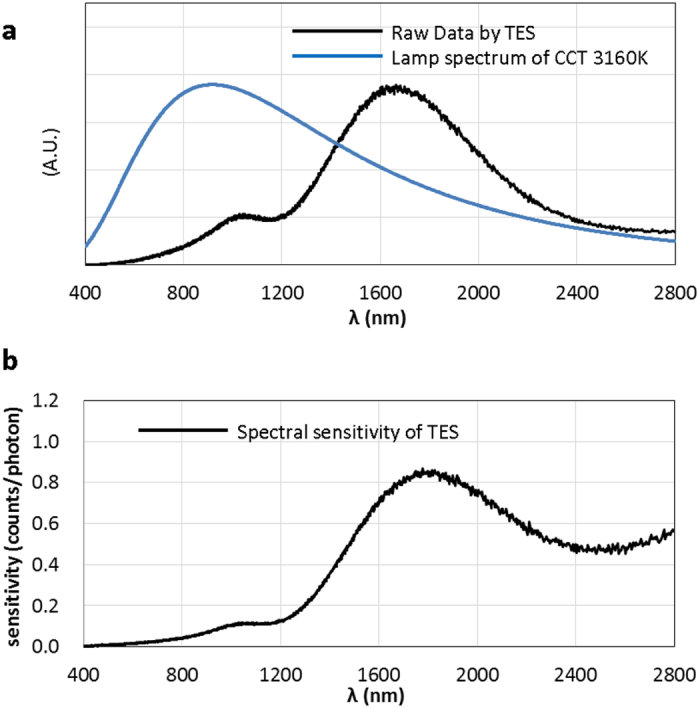
(**a**) Raw data of spectral measurement using TES (black line). Light from an incandescent lamp was introduced to the TES through an optical fiber. The correlated color temperature of the lamp was 3160 K, which determines the reference spectral distribution of the lamp (blue line). (**b**) Spectral sensitivity distribution of the TES.

Figure 2a shows the raw data spectrum of an incandescent lamp observed by the TES. The TES we used can detect photons with wavelengths from 400 to 2800 nm, which covers the visible to IR range. Because the background noise of the TES output voltage measurement (Fig. 1) is comparable to the signal height of incident low-energy photons with wavelengths longer than 2800 nm, this is taken to be the maximum wavelength detectable by the TES. Conventional spectrometers have difficulty measuring such a wide band of the spectrum simultaneously, because the sensitive regions of other photon detectors are not as wide as that of a TES. For example, PMTs are primarily sensitive to visible light, and even avalanche photodiodes for IR detection are only sensitive up to 1800 nm. Spectral elements also have problems with IR spectrometry because gratings cause multiple-order diffraction and prisms have less dispersion at longer wavelengths. Conversely, a TES has a wide band of spectral sensitivity from visible to near IR and is free from stray light and multiple-order diffraction noise.

The spectral sensitivity of the TES used in this study (Fig. 2b) was evaluated using the spectral distribution of an incandescent lamp, assumed from its correlated color temperature (CCT), which was determined from the lamp’s filament temperature, according to Planck’s law for black-body radiation. The CCT of the lamp was 3160 K. Its spectrum is shown in Fig. 2a. The TES used in this study showed less sensitivity in the visible light range because it was designed to detect the 1.5-μm telecommunication band^4^.

**Figure 3:**
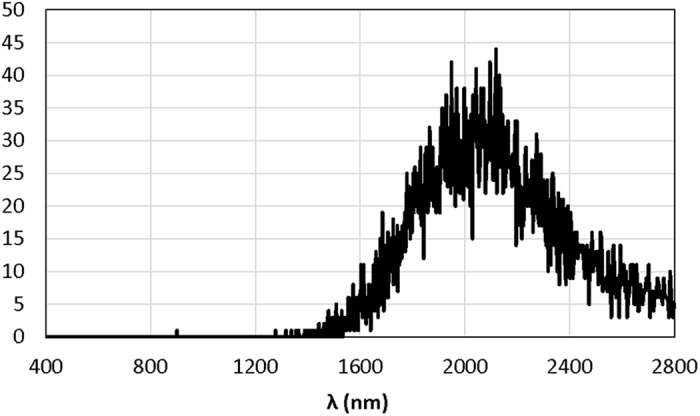
Background spectrum of TES detected for 500 s. There is no signal in the visible light region (360–830 nm). Signals detected over 1300 nm originate from black-body radiation at room temperature in accordance with Planck’s law.

Background observation by the TES through an optical fiber for a 500-s exposure period yielded no detected signals in the visible light region, whereas some signals were detected in the IR region (Fig. 3). This infrared signal originates from black-body radiation at room temperature^5^. In theory, at a finite temperature, all materials emit electromagnetic radiation, the spectrum of which depends only on the temperature and can be approximated by Planck’s law of black-body radiation. At room temperature, even without any illumination, all materials emit black-body radiation light, mainly in the IR range. The TES used in this study is so sensitive in the IR range that it detects black-body radiation even at room temperature. In the visible light region, however, the room-temperature black-body radiation is less than a single photon in thousand years from 1 m^2^. The single photon count detected at 900 nm shown in Fig. 3 may be from black-body radiation. Another possibility is two simultaneously detected incident photons with much longer wavelengths.

### Scanning microscope system using TES

**Figure 4:**
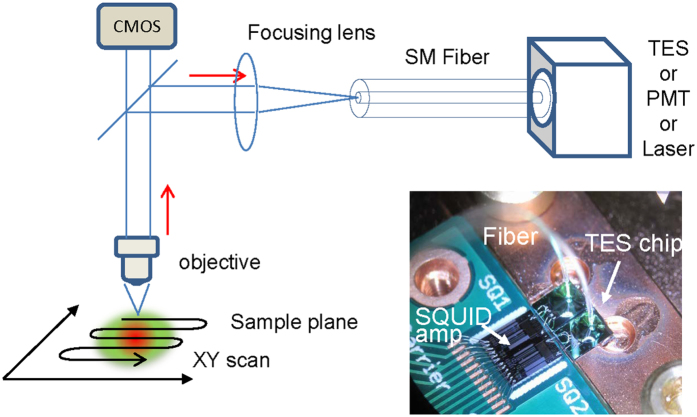
TES detector and scanning microscope optics. A test sample, a three-color ink-printed picture, was placed under an objective (×20), illuminated, and scanned on the XY stage. Collimated light through the objective was focused on the core at the end of the optical fiber by a focusing lens. The fiber core acts as a pinhole in confocal microscope systems. For comparison, TES and PMT were replaced with each other. A laser was employed in place of TES to focus the sample plane for observations by the CMOS camera. To illuminate visible-to-IR light, a sample plane was epi-illuminated by an incandescent lamp and side-illuminated by a blue LED.

To demonstrate the feasibility of a TES as a detector for spectral imaging, we constructed a scanning microscope system coupled with a TES as a detector via a single-mode optical fiber (Fig. 4). The core at the end of the fiber acts as a pinhole in the scanning confocal microscope^6^.

Because the size of the TES is on the order of several micrometers and it is placed in a refrigerator at 100 mK, light needed to be introduced via an optical fiber with a core diameter comparable to that of the TES. Therefore, the fiber optics were fabricated to combine the TES and the microscope optics. For comparison, a PMT was placed after the optical fiber. The sample was placed at the focal plane on an XY mechanical stage, epi- and side-illuminated, and scanned under a microscopy objective. Diffusively reflected light from a certain spot on the focal plane was collected and collimated by the objective and focused via a focusing lens on the core at the end of the optical fiber.

An image of the test pattern was built up in a point-wise fashion by scanning the spot across the focal plane and tiling the detected photon count. To depict a color image, the photon signals detected by the TES were discriminated by their wavelength as follows: photons shorter than 500 nm were blue, those from 500 to 600 nm were green, those from 600 to 800 nm were red, and those from 800 to 1500 nm were IR.

**Figure 5:**
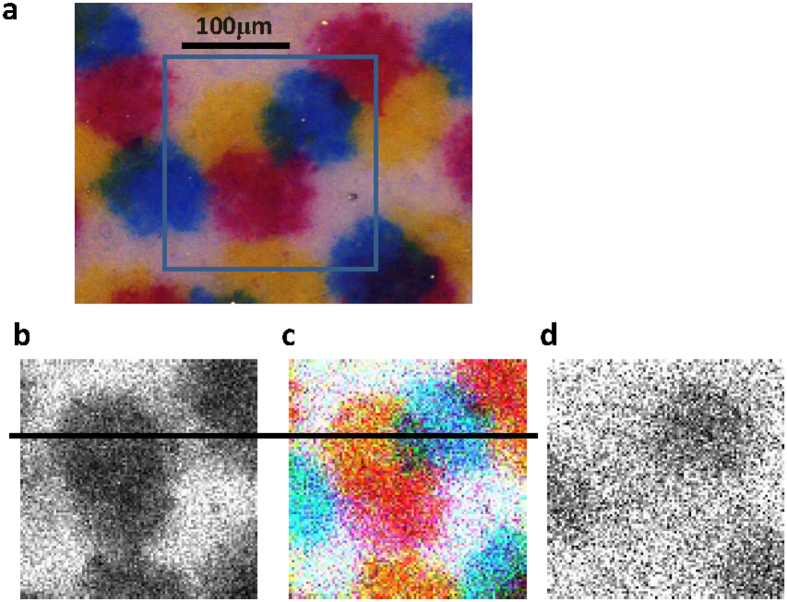
Images of a test target pattern sample with blue, yellow, and red ink spots. (**a**) Color CMOS image (1200 × 1600 pixels). The square indicates the area investigated by scanning using PMT and TES. The color CMOS image was obtained under a brighter illumination (×100) and at a longer exposure time (×10) conditions than those for PMT and TES. (**b**) PMT image (100 × 100 pixels) is only in gray scale, because PMT is not a spectral detector. (**c**) TES image of RGB color (100 × 100 pixels). All three color ink spots are clearly depicted. (**d**) TES image depicted using near-IR (800–1500 nm) signal (100 × 100 pixels). Under the illumination condition for PMT and TES, color CMOS could not detect any light signals.

As Fig. 5 shows, a test pattern with ink spots of three colors was observed. The images shown as Fig. 5b,c and d are 100 × 100 pixels. Each pixel spot size is 2 μm and is far wider than the areal resolution. Each spot was measured for 50 ms to count the incident photons and measure the wavelength of each photon. The total scanning time of 33 minutes comprised the TES exposure time (~8 min), the mechanical sample-stage action time, and computer data processing and system operation time. The areal resolution, defined as the diameter of the focused observed spot area, was 0.5 μm because the optical fiber core diameter was 10 μm and the objective was 20× . This value is comparable to that indicated by the Abbe diffraction limit theory:


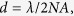


where *d* is the Abbe diffraction limit and *NA* is the numerical aperture. The *NA* of the objective used in this study was 0.4 and *λ* varied from 400 to 800 nm.

Under the dark illumination condition, the PMT provided a grayscale image in which it was difficult to distinguish red and yellow spots and difficult to find blue ink spots (Fig. 5b), because the spectral range of the PMT is limited. Even under the same illumination condition, the TES provided a smoother and better-contrasted RGB pattern (Fig. 5c). Furthermore, the near IR-range (800–1500 nm) photon count data at each spot provided a near-IR image (Fig. 5d). It is a great advantage of the TES that these imaging data sets are captured simultaneously.

**Figure 6:**
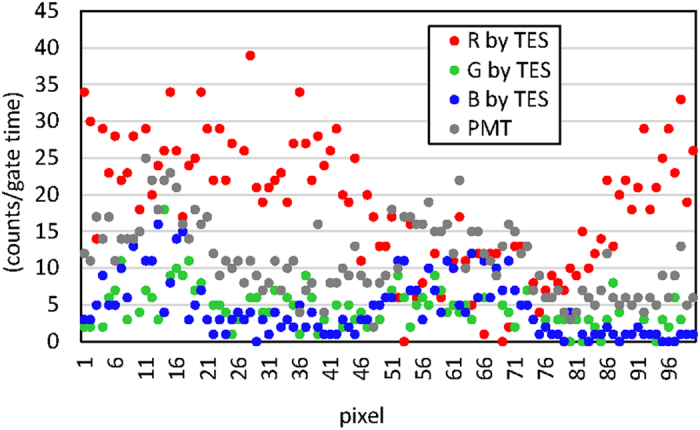
Raw data of the signal counts detected by the TES and PMT at the transection line on the images shown in Fig. 5b and c. The observed signal counts are well correlated with the patterns shown in Fig. 5.

The actual numbers of detected photons are significant. Figure 6 shows the raw data along the section line indicated in Fig. 5b and cas examples. The TES and PMT were able to detect comparable numbers of photon counts, although the TES used in this study was optimized for the 1.5-μm telecommunication band. The detection efficiency in the visible light region can be enhanced by redesigning the anti-reflection coating deposited on the TES.

The quality of the images is strongly correlated to S/N, given by the following equation:


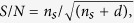


where *n*_*s*_ is the counted number of detected photons and *d* is the dark count. The noise in the photon count consists of the dark count and the signal shot noise, which is statistically modeled by a Poisson process. The exposure time for the images in Fig. 5 was 50 ms for both the TES and PMT, and the dark counts were zero and 0.5, respectively. At this short gate time, the S/N values for the TES and PMT depend primarily on the measured count values. If extremely dark samples are measured in conventional photon counting devices, the gate time needs to be much longer to accumulate larger count values. At longer gate times, the dark count level will influence the S/N values more than the signal count level.

### Spectrometry using TES in imaging application

**Figure 7:**
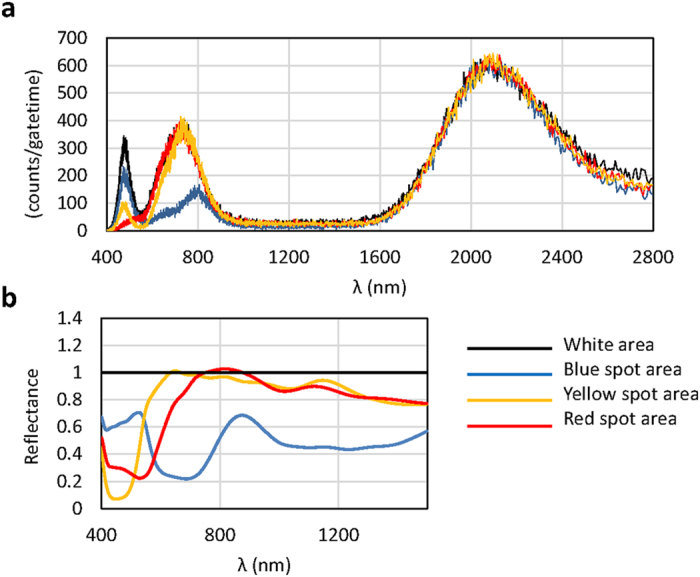
(**a**) TES spectral measurements. Spots in the different color areas on the test sample shown in Fig. 5 were observed. The exposure time was 200 seconds. The white area reflects most of the wavelength band of illumination, and each color spot absorbs its complementary color wavelengths. (**b**) Spectral reflectance of each color spot, normalized with respect to signals from a white color spot after smoothing.

A TES is more advantageous for longer gate times because of its extremely low background level, which is zero even for a gate time of 500 s (Fig. 3). Therefore, a TES can be used for highly sensitive spectral measurements from a selected focal area. A micro-fluidic chip is a suitable sample for such TES spectroscopy. For the imaging application, a user’s favorite focal area can be selected from an entire image, followed by measurement of the spectrum by TES. Figure 7 shows an example of TES spectral measurement using the test target pattern shown in Fig. 5. The raw data spectrum from the white area reflects the illumination light spectrum, and the spectrum from each color spot shows the absorbance of complementary color wavelengths (Fig. 7a). Spectral reflectance data (Fig. 7b) can be used to interpret imaging patterns in Fig. 5. For example, the blue ink spots absorb longer-wavelength photons and reflect blue light more than other colors. The blue spots are therefore shown less clearly by the PMT (Fig. 5b), which is less sensitive in the longer-wavelength range. In contrast, the TES images (Fig. 5c and d) shows blue spots because they absorb red to near-IR photons, whereas but other areas do not.

For scanning imaging purposes, however, longer gate times elongate the total scanning time. Therefore, a TES should be integrated and arrayed to shorten the capture time. As Fig. 2b shows, the sensitivity of a TES in the visible light region can be improved to allow a TES to accumulate more signals during a shorter gate time. In addition, the total scanning time can be reduced by optimizing the scanning system, e.g., by replacing the mechanical stage with a galvanometer mirror. These improvements can reduce the data accumulation time, making it possible to observe more dynamic samples, such as living cells. Although there remain some aspects of TES spectrometry to be improved, e.g. the fact that a TES has to be kept in a low-temperature refrigerator at 100 mK might restrict its applicability, we were successful in proving in this study that TES spectrometry is highly sensitive at the few-photon level and energy-dispersive in a wide wavelength range, from visible to IR. The results of this study indicate that a TES can contribute to applications in which important spectral imaging information, especially in the IR range, is required but cannot be obtained by more conventional methods.

## Conclusions

Few-photon color imaging using a TES as an energy-dispersive spectral photon-counting detector was successfully demonstrated. TES spectrometry is advantageous in spectral detection in the few-photon regime for longer gate times and also for near-IR detection. Additional improvements in TES technology, especially with regard to spectral sensitivity and array integration, would make TES’s useful as few-photon-counting spectral devices in a wider range of applications, such as bioimaging and nano-material analysis.

## Methods

### TES Single Photon Spectral Detector

The TES film was fabricated with superconducting/normal-metal bilayers (Ti/Au). The gold layer was made superconductive by the proximity effect, which lowered the superconducting transition temperature and determined the energy resolution. The transition temperature of titanium (0.4 K) was too high to resolve the energy of an optical photon. We optimized the thickness of the bilayers to achieve a desirable transition temperature. We chose gold because of its ease of fabrication. The effective sensitive area was 5 μm × 5 μm. The TES film was embedded in an optical cavity to enhance its photon absorption, i.e., its detection efficiency. A more detailed description of the fabrication process is provided in our previous report^4^.

The resistance change was read by a 16-series array (a superconducting quantum interference device, or SQUID) current amplifier that was placed close to the TES. An optical fiber (Nufern, UHNA7) with a mode field diameter of 3.2 μm and a fiber length of 30 cm was coupled with the micrometers of the TES. On the other end of the UHNA7 fiber, another optical fiber (Corning, SMF28) was connected using a fiber splicing method. The optical transmittance of the fiber connection, measured directly using a photo diode, was ~95%. The total system detection efficiency of the fiber-coupled TES was 85% at 1530 nm, based on our national standard for laser powers, as previously reported^4^.

The fiber coupled with the TES followed by the SQUID amplifier was placed on a cold stage in an adiabatic demagnetization refrigerator. The temperature on the cold stage was stabilized at 100 mK by regulating the magnetic current in a superconducting coil.

Optical photons were introduced into the TES via the optical fiber. The induced photon energy was absorbed in the TES, and the resultant TES resistance change was measured as voltage in the SQUID. The pulse height distribution of the SQUID voltage output was obtained by a multi-channel analyzer (MCA7600, SEIKO EG&G) or analyzed by two sets of single photon counters (SR400, Stanford Research Systems).

### Calibration

To obtain the wavelength distribution of the incident photons, the pulse height distributions were calibrated using a 1524-nm pulse laser (PLP-10–155, Hamamatsu, Japan) to irradiate multiplexed photons whose energies represented 750 nm (n = 2), 500 nm (n = 3), and 375 nm (n = 4) (Supplementary Figure 1).

As a reference spectrum, light from an incandescent lamp (Ushio 500 W, spectral irradiance standard lamp) was introduced to the TES through the optical fiber and measured to calibrate the spectral sensitivity. The spectrum of the incandescent lamp was determined by CCT at 3160 K, which is identical to the black-body radiation at this temperature.

### TES imaging optics

A scanning microscope system with an optical fiber was constructed as shown in Fig. 4. The optical fiber leading to the TES was 15 m in length and had a black cover. The effective core diameter and transmittance of the fiber were evaluated optically using an integrating sphere spectroradiometer and spectral irradiance standard lamp (Supplementary Figure 2). A He–Ne laser (Melles Griot, California, USA), a PMT (H18010a, Hamamatsu photonics, Japan), and a color complementary metal-oxide semiconductor (COMS) camera (1200Infinity 1–2 C, Lumenera Corp., Canada) were used to adjust and focus the optics and provide a detector comparison. The test sample was mechanically scanned using a motorized XY stage for a microscope (BIOS-105S, Optsigma, Japan). The stage control, data acquisition, and image processing were performed using LabVIEW (National Instruments, Texas, USA).

## Additional Information

**How to cite this article:** Niwa, K. *et al*. Few-photon color imaging using energy-dispersive superconducting transition-edge sensor spectrometry. *Sci. Rep.* **7**, 45660; doi: 10.1038/srep45660 (2017).

**Publisher's note:** Springer Nature remains neutral with regard to jurisdictional claims in published maps and institutional affiliations.

## Supplementary Material

Supplementary Figures
