# Supplementary material for: Few-photon color imaging using energy-dispersive superconducting transition-edge sensor spectrometry
Source: Sci Rep. 2017 Apr 4;7:45660. doi: 10.1038/srep45660 (PMC5379475; doi:10.1038/srep45660)
Supplement: Supplementary Figures [file srep45660-s1.pdf]

Supplementary Figures

Few-photon color imaging using energy-dispersive superconducting transition-edge sensor spectrometry

Kazuki Niwa, Takayki Numata, Kaori Hattori, Daiji Fukuda

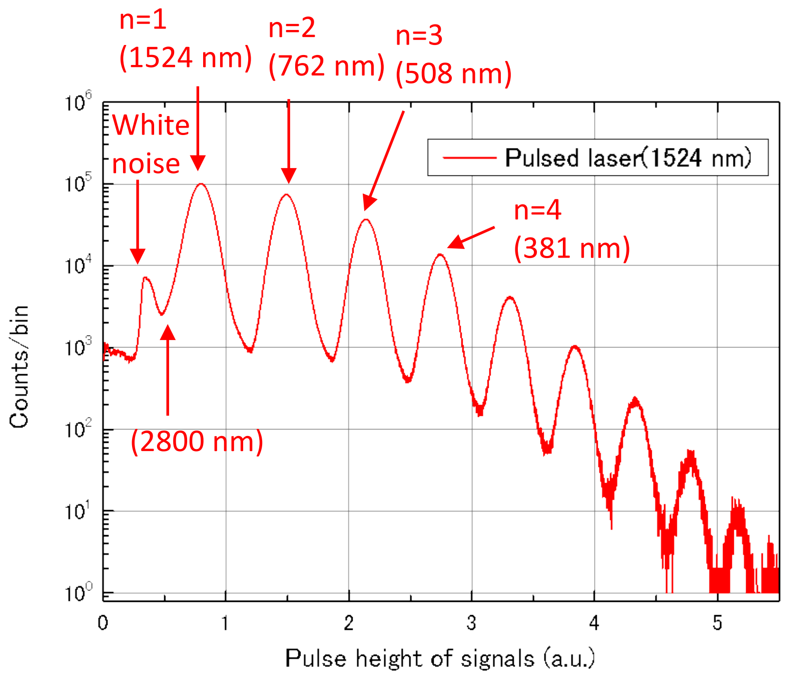

Supplementary Figure 1. Wavelength calibration of the TES using a pulsed laser (1524 nm). The number of pulses detected were plotted against the pulse height, similar to that in Figure 2b, 3, 7a. Each peak induced by the pulsed laser indicates the total energy of the multiplexed incident photons at the TES , which gives the correlation between the wavelength and pulse height. White noise signal appears as a peak where the photons have wavelengths longer than 2800 nm, which indicates the maximum wavelength limit.

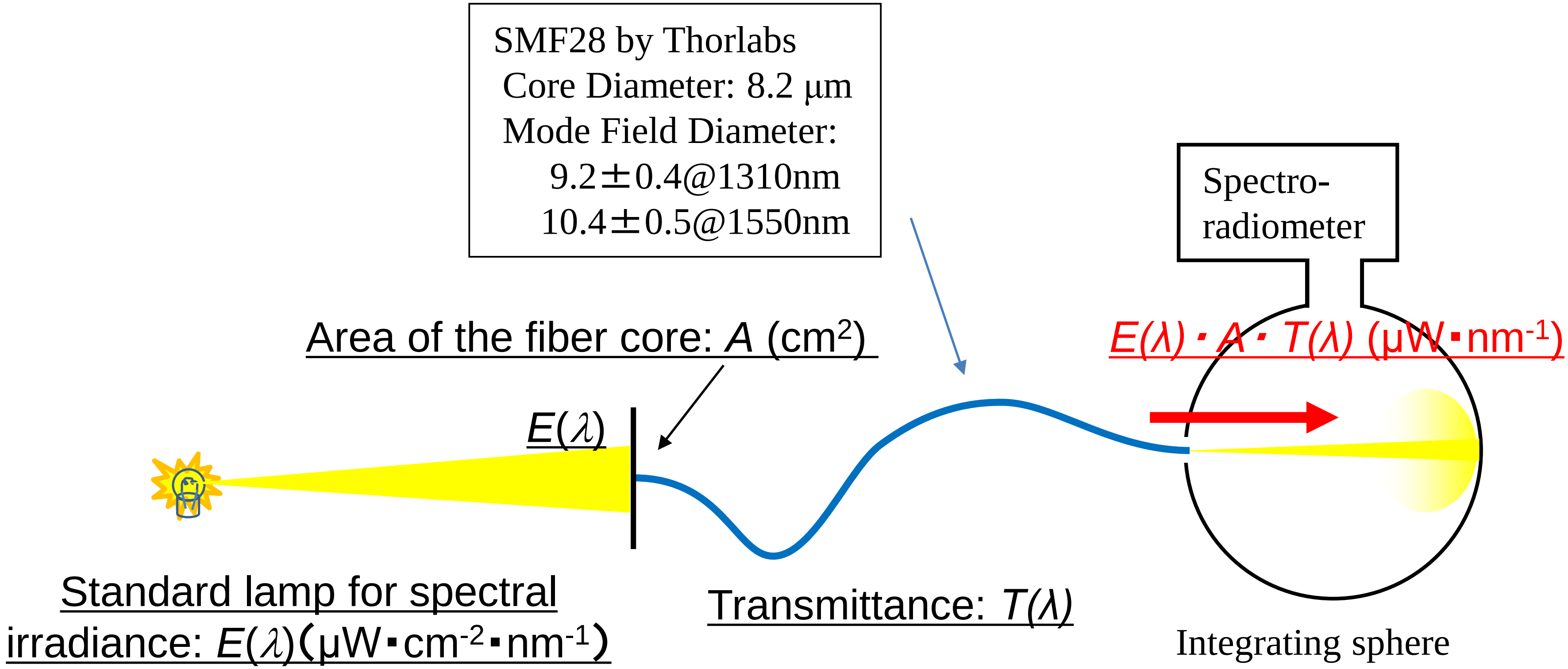

Supplementary Figure 2. Optics to evaluate the fiber core diameter, *A* (cm^2). The spectral irradiance value at the fiber end, *E(λ)* ($\mu W \cdot cm^{-2} \cdot nm^{-1}$), is provided on the standard lamp. The absolute spectral sensitivity of a spectro-radiometer with integrating sphere is calibrated using the same standard lamp and an aperture with a known area. The optically efficiency of the fiber, *A ･T(λ),* is calculated from the measured spectral flux ($\mu W \cdot nm^{-1}$), which is described as *E(λ) ･A ･T(λ).*
